# Trust predicts COVID-19 prescribed and discretionary behavioral intentions in 23 countries

**DOI:** 10.1371/journal.pone.0248334

**Published:** 2021-03-10

**Authors:** Stefano Pagliaro, Simona Sacchi, Maria Giuseppina Pacilli, Marco Brambilla, Francesca Lionetti, Karim Bettache, Mauro Bianchi, Marco Biella, Virginie Bonnot, Mihaela Boza, Fabrizio Butera, Suzan Ceylan-Batur, Kristy Chong, Tatiana Chopova, Charlie R. Crimston, Belén Álvarez, Isabel Cuadrado, Naomi Ellemers, Magdalena Formanowicz, Verena Graupmann, Theofilos Gkinopoulos, Evelyn Hye Kyung Jeong, Inga Jasinskaja-Lahti, Jolanda Jetten, Kabir Muhib Bin, Yanhui Mao, Christine McCoy, Farah Mehnaz, Anca Minescu, David Sirlopú, Andrej Simić, Giovanni Travaglino, Ayse K. Uskul, Cinzia Zanetti, Anna Zinn, Elena Zubieta

**Affiliations:** 1 Department of Neuroscience, Imaging and Clinical Science, University of Chieti-Pescara, Chieti, Italy; 2 Department of Psychology, University of Milano-Bicocca, Milan, Italy; 3 Department of Political Sciences, University of Perugia, Perugia, Italy; 4 Department of Psychology, Monash University, Clayton, Malaysia; 5 Digital Human-Environment Interaction Lab, Universidade Lusòfona, Lisbon, Portugal; 6 Department of Psychology, Eberhard Karls Universität Tübingen, Tübingen, Germany; 7 Department of Psychology, Université de Paris, Paris, France; 8 Department of Psychology and Education Sciences, University Alexandru Ioan Cuza, Iasi, Romania; 9 Faculty of Social and Political Sciences, University of Lausanne, Lausanne, Switzerland; 10 Department of Psychology, TOBB University of Economics and Technology, Ankara, Turkey; 11 Department of Psychology, Utrecht University, Utrecht, The Netherlands; 12 School of Psychology, University of Queensland, Brisbane, Australia; 13 Department of Psychology, University of Almería, Almeria, Spain; 14 University Social Sciences and Humanities, Ho Chi Minh, Poland; 15 School of Psychology, Nicolaus Copernicus University, Torun, Poland; 16 Department of Psychology, DePaul University, Chicago, Illinois, United States of America; 17 Faculty of Education, Greenwich University, London, United Kingdom; 18 Department of Psychology, University of Limerick, Limerick, Ireland; 19 Faculty of Social Sciences, University of Helsinki, Helsinki, Finland; 20 Southwest Jiaotong University, Chengdu, China; 21 School of Humanities and Social Science, Universidad del Desarrollo, Santiago, Chile; 22 The Chinese University of Honk Kong, Honk Kong, China; 23 School of Psychology, University of Kent, Kent, United Kingdom; 24 Department of Psychology, University of Exeter, England, United Kingdom; 25 Faculty of Psychology, University of Buenos Aires, Buenos Aires, Argentina; Universidad Nacional de Educacion a Distancia, SPAIN

## Abstract

The worldwide spread of a new coronavirus (SARS-CoV-2) since December 2019 has posed a severe threat to individuals’ well-being. While the world at large is waiting that the released vaccines immunize most citizens, public health experts suggest that, in the meantime, it is only through behavior change that the spread of COVID-19 can be controlled. Importantly, the required behaviors are aimed not only at safeguarding one’s own health. Instead, individuals are asked to adapt their behaviors to protect the community at large. This raises the question of which social concerns and moral principles make people willing to do so. We considered in 23 countries (*N* = 6948) individuals’ willingness to engage in prescribed and discretionary behaviors, as well as country-level and individual-level factors that might drive such behavioral intentions. Results from multilevel multiple regressions, with country as the nesting variable, showed that publicized number of infections were not significantly related to individual intentions to comply with the prescribed measures and intentions to engage in discretionary prosocial behaviors. Instead, psychological differences in terms of trust in government, citizens, and in particular toward science predicted individuals’ behavioral intentions across countries. The more people endorsed moral principles of fairness and care (vs. loyalty and authority), the more they were inclined to report trust in science, which, in turn, statistically predicted prescribed and discretionary behavioral intentions. Results have implications for the type of intervention and public communication strategies that should be most effective to induce the behavioral changes that are needed to control the COVID-19 outbreak.

## Introduction

The worldwide spread of a new coronavirus (SARS-CoV-2) since December 2019 has posed a severe threat for individuals’ health and well-being [1]. Indeed, at the time of writing this piece (February, 2021), around 107 million people worldwide have been infected, and more than 2,350,000 individuals have died due to the novel coronavirus disease (COVID-19). Researchers across the world have joined forces to determine key characteristics of the virus, optimize treatment of patients, and find a vaccine. Recently, several vaccines have been released, and Governments have started the inoculation procedures. Nevertheless, until the effectiveness of the vaccines will be visible and/or effective medical treatments are found, it is only through behavioral change that people can counter the spread of the virus such as washing hands more frequently, avoiding traveling and congregating in public places, keeping a distance from others, as well as starker measures such as self-isolation and quarantine [2]. These behaviors, which have been prescribed by local governments throughout the world, aim to safeguard not just the health of single individuals but also of their communities at large.

Because of these measures, the pandemic has also reshaped social life, and behavioral scientists have applied their knowledge to analyze the broader impact of these changes for individual well-being and public health [3–6]. Here, we extend prior efforts by focusing on a key factor in current endeavors to reduce the spread of the virus and contain the number of deaths, namely the willingness of citizens in different countries to change their behavior to limit the spread of COVID-19. To this end, we compare behavioral intentions relating to the spread of COVID-19 across 23 countries and examine factors that may account for differences across these countries in people’s willingness to adapt their behavior. Such an examination can offer crucial insights to control the COVID-19 outbreak.

The success of current measures cannot be guarded by relying on compliance with prescriptive measures [5,7,8]. The continuous nature and sheer extent of the required changes—which should also be implemented in people’s homes and private spaces—makes monitoring impossible for authorities. This is why individual support and willingness to cooperate—which are not prescribed behaviors—play a key role in overcoming the pandemic [4,9–11]. Indeed, despite media reports of public selfishness (e.g., people stockpiling scarce resources), there is also considerable evidence of widespread pro-social and discretionary behaviors (e.g., donating to charities to help them fight the disease; buying groceries and supplies for people who are in quarantine). Such discretionary, or extra-role behaviors go beyond mandated behaviors prescribed by authorities or explicit regulations, and involve a voluntary effort that, although not required, helps the whole group or community to deal more effectively with the pandemic. Therefore, assessing people’s willingness to engage in such discretionary behaviors—beyond their compliance with prescribed behaviors—is an important indicator of the likelihood that behavioral changes will be made. Here, we aim at testing the psychological factors that might promote those prescribed and discretionary behaviors by adopting a cross-cultural approach. Specifically, we tested whether the psychological experience of trust (toward the government, the fellow citizens, and science) has a greater predictive power than that the publicized statistics of the pandemic in terms of infections and deaths to foster behavioral change.

### Theoretical background and hypotheses

Despite the importance of engaging in COVID-19 prescribed and discretionary behaviors in the management of the pandemic, only few studies have addressed the factors driving such behaviors and most of them have almost exclusively focused on prescribed behaviors neglecting discretionary and non-mandatory behaviors [e.g., 5,8,9,12]. Extending prior work, here we compare the willingness to engage in prescribed and discretionary behaviors across different countries by considering two possible predictors.

First, we address the severity that the pandemic is posing to public health of various national communities and individuals within these communities in terms of number of victims, as communicated by the Authorities. This reflects current public communications policies transmitting this type of information as a primary strategy to convince people that behavioral changes are needed. Indeed, it has been argued that perceived personal vulnerability connected with the spread of the virus in their community, might induce individuals to implement behavioral changes needed to contain the virus spread [13]. It has also been argued that the severity of the pandemic communicated by information news about daily infections and deaths rates in a given country might foster virtuous behaviors among its citizens [14,15].

Second, we assess individual experiences of trust, which are known to predict willingness to coordinate efforts with others and cooperate with requests from authorities [16]. In this sense, we follow up on prior speculations about the possible impact of intercultural differences. These have suggested that cultural ‘tightness’ versus ‘looseness’ accounts for cross-national differences in compliance with requests from authorities, and that ‘individualism’ versus ‘collectivism’ explains cross-national differences in willingness to coordinate efforts with other individuals [6,17]. We address trust as the most proximal psychological variable that is relevant in determining individual level responses across and within different national contexts. We distinguish between trust in three types of actors that are relevant to the required behavioral changes [18]: the government which imposes the required changes and requests citizen’s compliance, fellow citizens whose cooperation is needed for individual efforts to be effective, and science as the source of information arguing that these changes are needed.

Trust in governments has long been viewed as an important determinant of citizens’ compliance with public health policies, restrictions and guidelines [19]. For example, research during the 2014–15 Ebola outbreak in Liberia has shown that enhancing trust in health officials increases the willingness of citizens to follow and respect public health measures [19,20]. As a case in point, it has been shown that trust toward government increased compliance with governmental rules related to COVID-19 amongst Italian and French individuals, while the absence of trust in conjunction with low self-concern regarding the virus significantly reduced compliance [12]. On the other hand, a paradoxical effect of trust in government has also been found, such that high levels of trust in the government resulted in the underestimation of risk and non-compliant behavior, because of reduced perceived need to take individual action to control the risks [21]. Thus, trust in government and institutions may be an important antecedent of increasing compliance with governmental demands and regulations [22,23], and it is worth investigating how it impacts individuals’ behaviors related to COVID-19.

In situations that require interpersonal coordination to optimize collective outcomes, trust in others has been identified as an important predictor of the willingness to cooperate [24,25]. The belief that most people can be trusted (i.e., the belief that people are likely to be reliable, cooperative and benevolent) has been found to be particularly relevant to adherence to health recommendations [26]. Indeed, research concerning health decision-making has shown that trust toward fellow citizens is associated with prosocial behavioral intentions, such as willingness to get vaccinated [27–29; but see also for different results, 30].

Scientists and their knowledge are the key source of public advice about behavioral changes that are needed to curb the spread of COVID-19 [8]. Although scientists should have a legitimate power of influence granted by their expertise, in recent decades we have witnessed an increasing divide between science and society, corresponding with rising levels of distrust in science in large parts of the world [31–33]. This is consequential, as research suggests that distrust in science -often underpinned by conspiracy beliefs [34] and specific ideologies [35]- affects health-related attitudes and behaviors [e.g., vaccination, attitudes toward HIV; 36,37]. Along similar lines, we proposed that individuals who distrust science might be less motivated to follow science-based advice about important behavioral guidelines to curb the pandemic.

To examine these issues, we investigated people’s willingness to comply with prescribed measures and discretionary efforts to manage the pandemic in 23 different countries across the world, that include a range of national, political, and cultural contexts. The countries also differed from each other in terms of the reported spread of the COVID-19 virus, which allowed us to have high variability in terms of trust as well as in the actual number of infections and deaths. In a multilevel approach, we examined whether the communicated severity posed by COVID-19, in terms of publicized numbers of infections and deaths in the different countries represents a possible predictor of behavioral intentions. We compared this to the predictive value of trust in government, fellow citizens, and science to account for differences in behavioral intentions in response to the pandemic. Indeed, trust in different agents might be central to promote behavioral changes aimed at preserving public health. Differently, a climate of suspicion might undermine the motivation to follow the health agencies advise and compliance with guidelines aimed at controlling the pandemic. Based on this rationale, we anticipated that individuals’ behavioral intentions would be better predicted by trust toward government, fellow citizens, and in particular science and scientists than by actual threat—that is, in terms of infections and deaths (H1).

We further explored the origins of trust, by examining endorsement of moral values that feed trust in specific actors [38–40]. It has been argued that epidemics represent a category of disease that not only confront our own mortality, but also force us to consider the moral relations that we have with others in society [41]. We built our theorizing on Moral Foundation Theory [42–44] which distinguishes between two clusters of moral values. Loyalty, Authority, and Purity are seen as ‘binding’ foundations, and emphasize the importance of preserving communities by means of obedience, duty and protection of cultural boundaries [45]. Care (vs harm) and Fairness are indicated as ‘individualizing’ foundations, that primarily refer to the importance of protecting other individuals, by showing compassion and defending civic liberties.

Prior research suggests that trust in specific actors is associated with the endorsement of different moral foundations. For instance, trust in institutions tends to relate to the endorsement of binding values and priorities (protection, respect for authority, and responsibility), both at the individual [46,47] and country level [48]. Likewise, moral concerns about purity predict trust in science [49; see also 50,51]. Thus, prior research suggests that the origins of individual’s perception of social agencies and institutions, the willingness to accept their suggestions, and the resultant behavioral responses in terms of support, cooperation and compliance should be understood in the context of their moral worldviews.

In the context of the present research, we note that specific groups and cultures prioritize binding versus individualizing moral foundations to a different extent [e.g., WEIRD—Western, Educated, Industrialized, Rich and Democratic—vs. non-WEIRD countries; 52]. The inclusion of a range of different countries in the current research helped us to capture such cross-country variability in the endorsement of moral foundations. Based on the rationale described above, we examined whether the extent to which people in different countries prioritize these distinctive moral principles may have far reaching diverging consequences on their trust toward government, fellow citizens, and science. We expected endorsement of the binding moral foundations to be positively related to trust in government and fellow citizens, and negatively related to trust in science (H2a). In contrast, we expected endorsement of the individualizing moral foundations to be positively related to trust in science and negatively related to trust in government and fellow citizens (H2b). Thus, it might follow that trust would operate as a mediating mechanism of the hypothesized relationship between moral principles and behavioral intentions (H3).

## Method

The research reported in the manuscript has been approved by the local ethics committee of the University of Milano-Bicocca (RM-2020-271). At the beginning of the survey, participants were provided with a description about the study (e.g., methods, institutional affiliations of the PI) and were informed of their right to refuse to participate in the study or withdraw consent to participate at any time during the study without reprisal. They then confirmed that they properly understood the instructions, gave their written consent, and moved on to completing the study. The study was conducted in accordance with the guidelines defined by the Declaration of Helsinki.

### Participants

We collected data in 23 countries that vary along the collectivism -individualism and tightness-looseness dimensions (Gelfand, et al., 2011): Argentina (*N* = 260), Australia (*N* = 303), Bangladesh (*N* = 304), Bosnia (*N* = 238), Chile (*N* = 319), China (*N* = 397), Finland (*N* = 300), France (*N* = 239), Germany (*N* = 352), Greece (*N* = 299), Ireland (*N* = 316), Italy (*N* = 350), Malaysia (*N* = 179), Netherlands (*N* = 320), Poland (*N* = 314), Romania (*N* = 381), Russia (*N* = 317), South Korea (*N* = 130), Spain (*N* = 320), Switzerland (*N* = 351), Turkey (*N* = 300), UK (*N* = 300), and US (*N* = 359). The total sample comprised 6,948 participants (3,806 women, 2,785 men, 85 non-binary or other, 272 missing; *M*_age_ = 34.22, *SD*_age_ = 15.13) who were recruited using convenience sampling by distributing the survey on social networking sites, and/or via Prolific Academic. S1 Table in S1 File (see S1 File) provides demographic information for each sample.

### Measures and procedure

Data were collected between April, 10^th^ and May, 19^th^ 2020. Participants provided socio-demographic information, and details about their own COVID-19 symptoms and experiences. Participants responded to all questions on a 7-point scale from 1 (not at all) to 7 (very much). Participants were then presented with the Moral Foundations Questionnaire (MFQ; 30 items; MFQ-Individualizing α = .77, MFQ-Binding α = .85; 42) and read a message about the necessity to adhere to the COVID-19 prevention measures of social distancing. Specifically, we asked participants to read one of two messages that were similar in length, wording and structure, but were framed slightly differently. The message did not influence participants responses to the main DVs, so this was not included in the subsequent analyses (see S4 Table in S1 File). We asked participants two questions about the valence of the article and five questions about its perceived relevance to each of the moral foundations (see S1 File for additional measures that are not included in the current paper).

Participants then indicated their perceived trust toward their national government (e.g., *I trust our Government’s competence in the management of the COVID-19 crisis*), toward fellow citizens (i.e., *I trust that other [country] citizens will respect the prescriptions imposed to avoid contagion*), and science (e.g., *We should trust the work of scientists*). After that, participants completed 14 items about their willingness to comply with the prescribed COVID-19 prevention behaviors (7 items, e.g., *Whenever it is possible*, *self-isolating at home*; α = .82), as well as their intention to display discretionary COVID-19 behaviors (7 items, e.g., *Depending on our ability*, *all of us should give money to charities to help them fight the disease*; α = .68) related to the management of the pandemic. The survey was translated and back-translated to the different languages by members of the research team. The survey included additional scales (see S2 Table in S1 File for descriptive statistics by country) (see the S1 File for a complete list of measures). As country-level variables, we used COVID-19 deaths per million people—assessed during the data collection time within each country (data source: https://www.worldometers.info)–and the Gini coefficient, a widely adopted measure of economic inequality.

## Results

### Descriptive statistics

**Fig 1:**
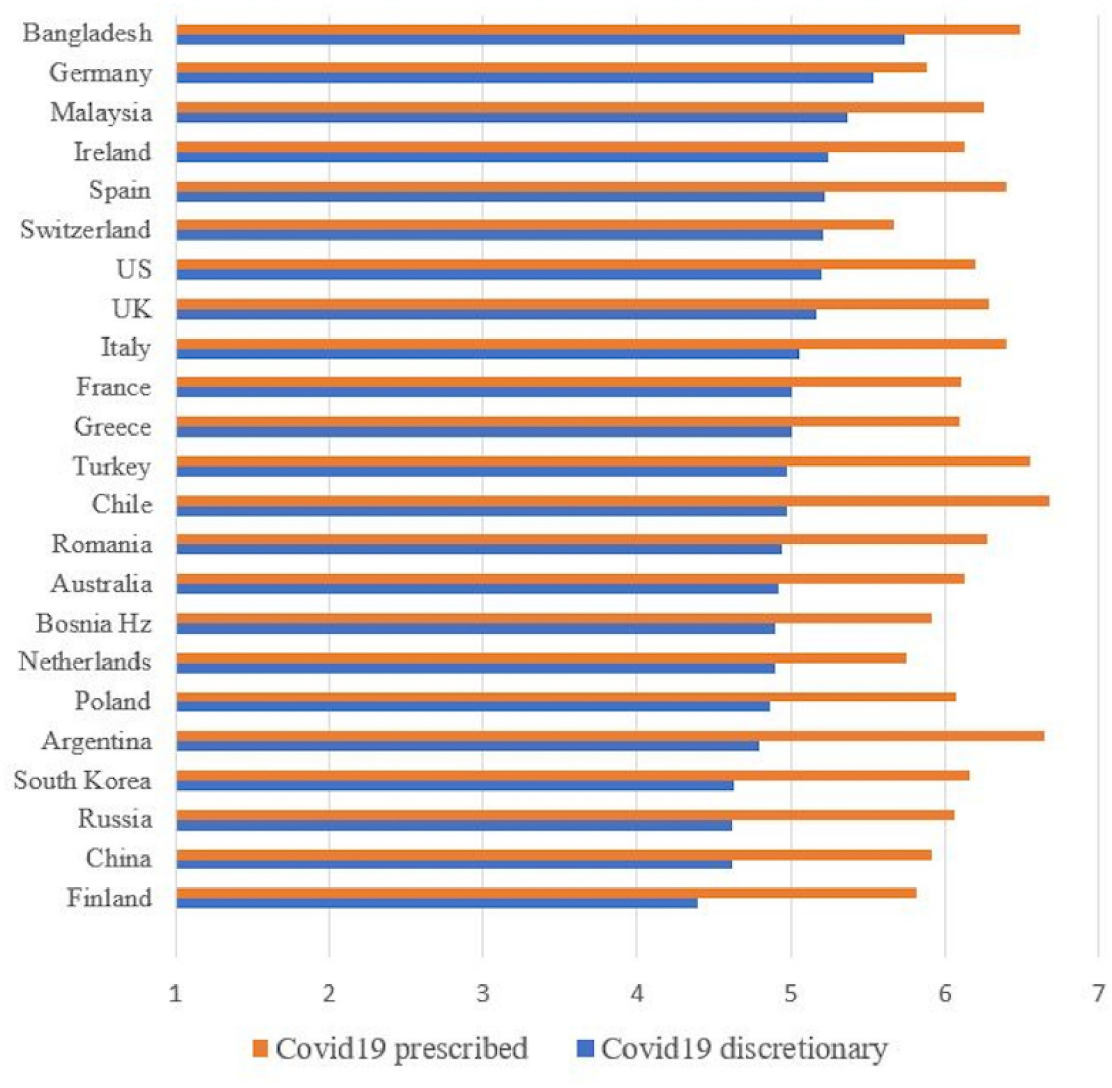
Mean levels of declared compliance to prescribed behaviors and willingness to display discretionary behaviors by country. Countries in the figures are ranked in a descending way according to discretionary behavioral intentions mean levels.

**Fig 2:**
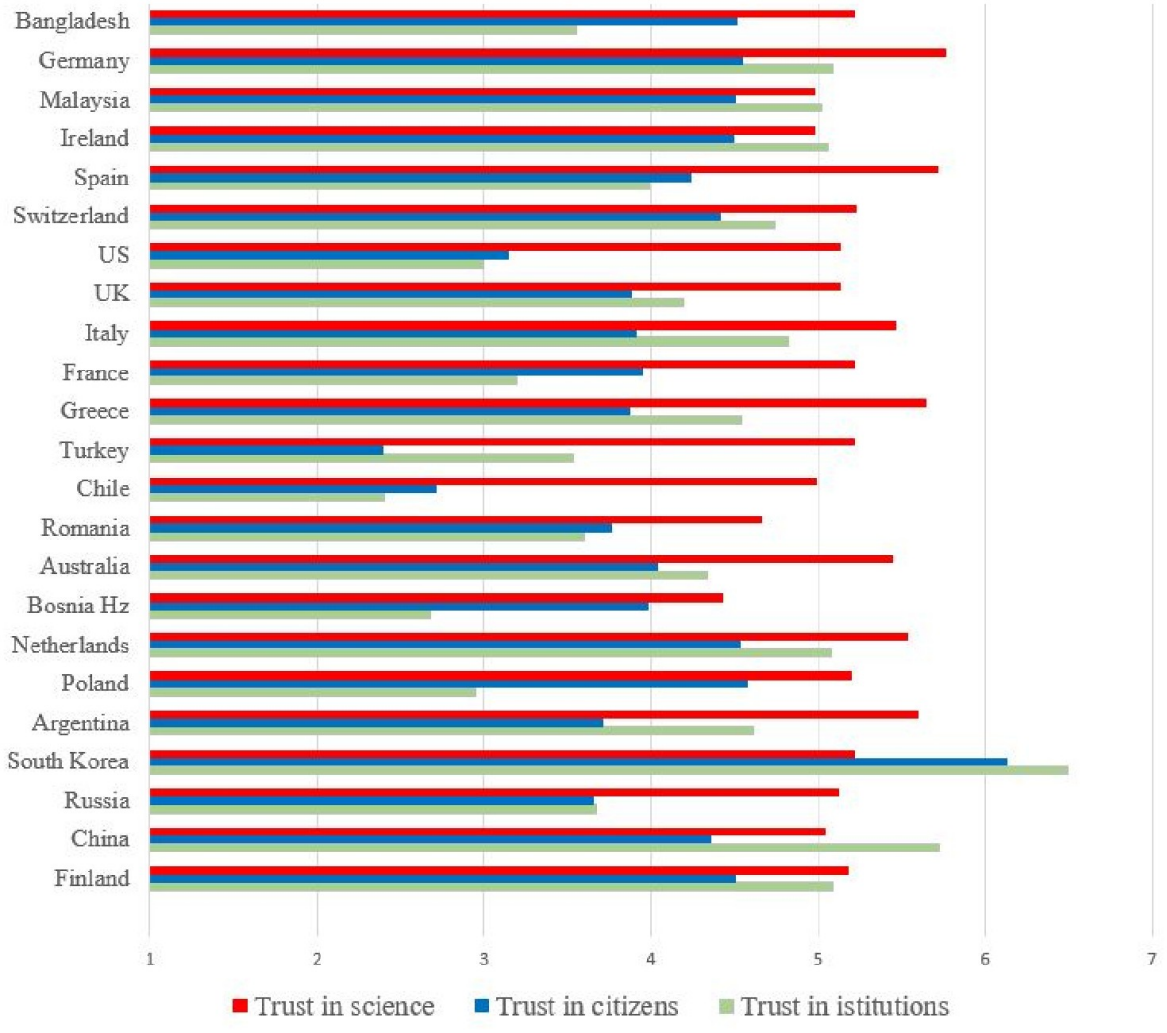
Mean levels of trust in science, citizens and government by country.

Fig 1 shows the mean levels of compliance with prescribed behaviors, as well as the willingness to engage in discretionary behaviors by country. As the figure shows, the rank order differs according to intention to engage in prescribed versus discretionary behaviors. Fig 2 shows the mean levels of trust in institutions, citizens and science by country.

### Multivariate analyses

**Fig 3:**
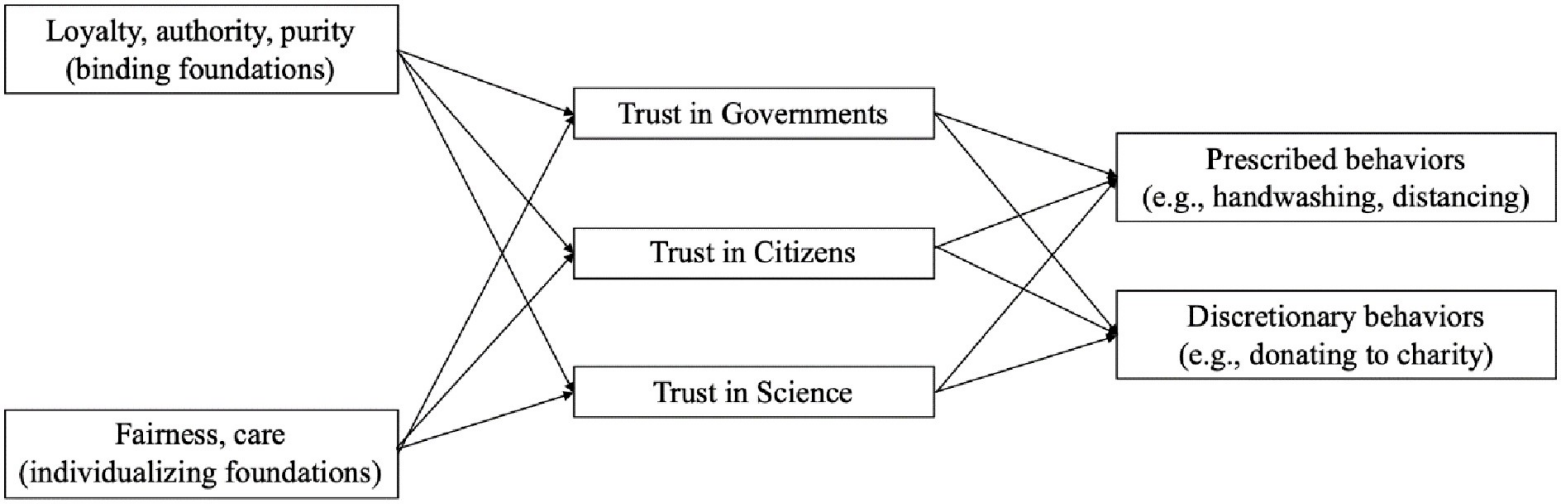
Hypothesized theoretical model.

We conducted multi-level path analysis in Mplus version 8.8 [53]. This type of analysis allowed us to take into account the nested structure of the data collected across 23 different countries. Fig 3 depicts the tested mediation model at the individual level (Level 1). This model included the individualizing and binding moral foundations as predictors of mandatory and discretionary Covid-19 related behaviors via the mediating role of trust in government, trust in citizens, and trust in science. The intraclass correlation coefficient for mandatory behaviors (.084) and for discretionary behaviors (.080) indicated that less than 10% of the variance for prescribed and discretionary behavior was attributed to the country variable.

**Fig 4:**
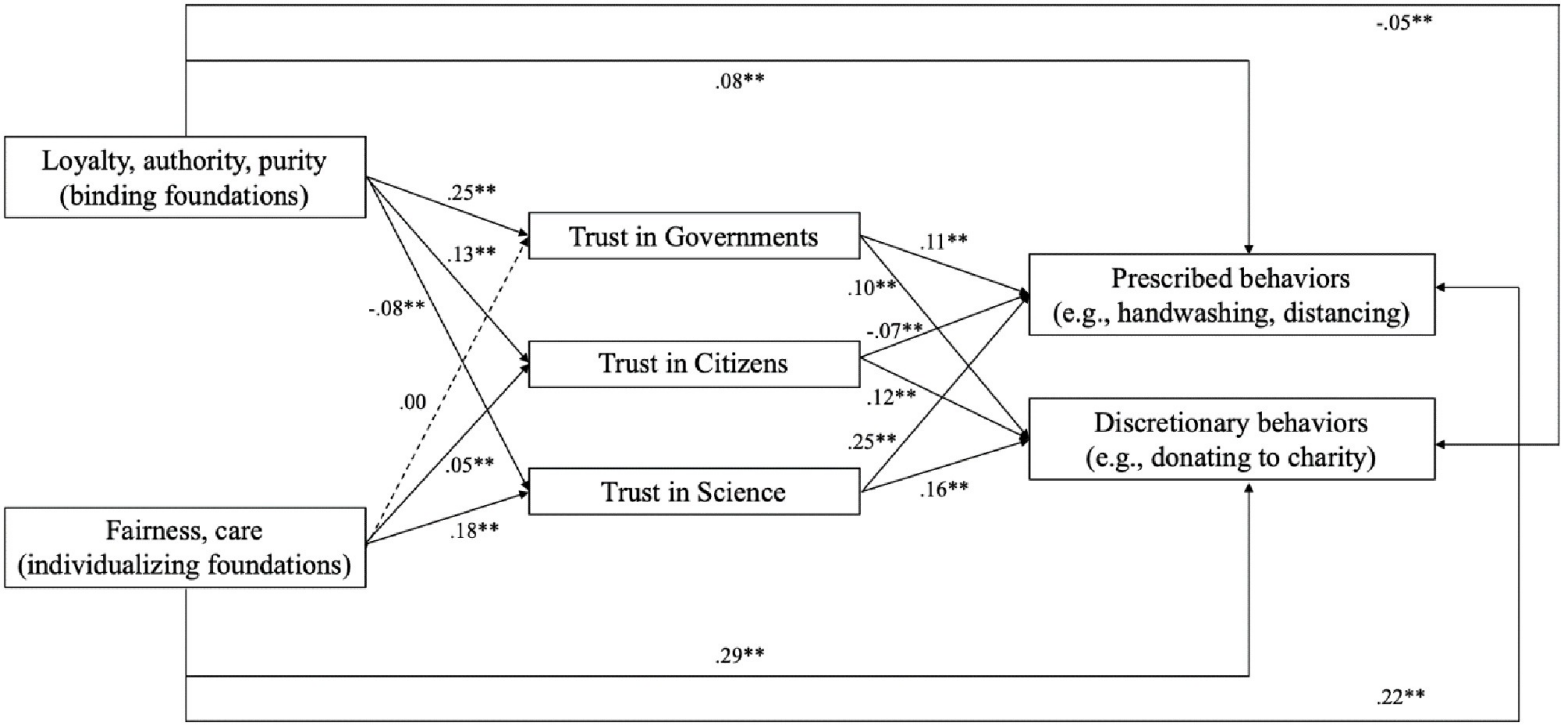
Multivariate multi-level regression model in which individualizing moral foundations (fairness and care) and binding moral foundations (loyalty and authority) were modelled as independent variables, prescribed and discretionary COVID-19 related behaviors as dependent variables, and trust in governments, trust in citizens, and trust in science as parallel mediators. **p < .01.

We performed the analyses in three steps. In step 1, the simple mediation model was tested. In step 2, a series of individual (Level 1) control variables (age, gender, education, community size, and political orientation) were entered into the model. In step 3, we included relevant country level control variables (Level 2; Gini coefficient and COVID-19 deaths per million people. We repeated the analyses with number of infections per million people, and the results were almost identical). Fig 4 graphically depicts the main results. As shown in Table 1, in step 1, the binding moral foundations significantly and positively predicted trust in government, and in fellow citizens, and negatively predicted trust in science (H2a). In contrast, the individualizing moral foundations positively predicted trust in fellow citizens, and trust in science (H2b). All the predictors and mediators were significantly associated with prescribed and discretionary behaviors (see Table 1 for regression parameters and S3 Table in S1 File for residual variances).

**Table 1:** Estimated parameters of the three models tested. Step 1 is the model depicted in Fig 1, Step 2 includes the individual level control variables, step 3 includes the country-level variables.

	Step 1		Step 2		Step 3			
					*Within Level*		*Between Level*	
	β [95% CI]	*p*	β [95% CI]	*p*	β [95% CI]	*p*	β [95% CI]	*p*
**Trust in government**								
Individualizing	0.00 [-0.091, 0.088]	.971	-0.01 [-0.092, 0.077]	.82	0.00 [-0.060, 0.068]	.87		
Binding	0.27 [0.120, 0.412]	< .01	0.27 [0.133, 0.405]	< .01	0.25 [0.160, 0.344]	< .01		
Age	--	--	0.02 [-0.036, 0.074]	.37	0.02 [-0.036, 0.072]	.39		
Gender	--	--	-0.01 [-0.050, 0.032]	.57	-0.01 [-0.051, 0.033]	.58		
Education	--	--	0.02 [-0.021, 0.057]	.24	0.02 [-0.020, 0.060]	.19		
Community size	--	--	0.03 [-0.022, 0.087]	.12	0.03 [-0.020, 0.083]	.11		
Political orientation	--	--	-0.03 [-0.151, 0.095]	.56	0.02 [-0.132, 0.175]	.72		
Gini	--	--	--	--			-0.58 [-1.172, 0.022]	< .05
Deaths per million people							0.19 [-0.367, 0.743]	.38
**Trust in citizens**								
Individualizing	0.05 [0.010, 0.099]	< .01	0.04 [0.001, 0.087]	< .01	0.05 [0.004, 0.093]	< .01		
Binding	0.15 [0.074, 0.226]	< .01	0.14 [0.069, 0.215]	< .01	0.13 [0.063, 0.205]	< .01		
Age	--	--	0.12 [0.039, 0.200]	< .01	0.12 [0.039, 0.200]	< .01		
Gender	--	--	0.00 [-0.048, 0.042]	.88	0.00 [-0.048, 0.042]	.86		
Education	--	--	0.04 [-0.014, 0.085]	.07	0.04 [-0.013, 0.085]	.06		
Community size	--	--	0.00 [-0.023, 0.027]	.84	0.00 [-0.023, 0.027]	.83		
Political Orientation	--	--	0.00 [-0.033, 0.032]	.94	0.01 [-0.034, 0.057]	.53		
Gini	--	--	--	--			-0.75 [-1.259, -0.245]	< .01
Deaths per million people							0.14 [-0.307, 0.583]	.42
**Trust in science**								
Individualizing	0.20 [0.152, 0.253]	< .01	0.19 [0.139, 0.237]	< .01	0.18 [0.130, 0.225]	< .01		
Binding	-0.12 [-0.191, -0.049]	< .01	-0.10 [-0.163, -0.038]	< .01	-0.08 [-0.147, -0.020]	< .01		
Age	--	--	-0.02 [-0.074, 0.031]	.29	-0.02 [-0.073, 0.032]	.32		
Gender	--	--	0.02 [-0.031, 0.077]	.27	0.02 [-0.030, 0.079]	.25		
Education	--	--	0.08 [0.041, 0.112]	< .01	0.08 [0.043, 0.114]	< .01		
Community size	--	--	0.01 [-0.059, 0.072]	.80	0.01 [-0.055, 0.076]	.68		
Political orientation	--	--	-0.07 [-0.110, -0.024]	< .01	-0.07 [-0.120, -0.024]	< .01		
Gini	--	--	--	--			-0.24 [-0.798, 0.320]	.27
Deaths per million people							0.30 [-0.152, 0.746]	.09
**Prescribed behaviors**								
Individualizing	0.23 [0.184, 0.270]	< .01	0.22 [0.183, 0.264]	< .01	0.22 [0.173,0.263]	< .01		
Binding	0.07 [0.033, 0.112]	< .01	0.07 [0.037, 0.107]	< .01	0.08 [0.044, 0.119]	< .01		
Trust in government	0.11 [0.068, 0.154]	< .01	0.11 [0.067, 0.157]	< .01	0.11 [0.066, 0.157]	< .01		
Trust in citizens	-0.07 [-0.114, -0.017]	< .01	-0.07 [-0.114, -0.021]	< .01	-0.07 [-0.116, -0.022]	< .01		
Trust in science	0.25 [0.207, 0.290]	< .01	0.25 [0.210, 0.286]	< .01	0.25 [0.209,0.284]	< .01		
Age	--	--	0.02 [-0.023, 0.069]	.20	0.02 [-0.023, 0.071]	.19		
Gender	--	--	-0.05 [-0.093, -0.011]	< .01	-0.05 [-0.094, -0.011]	< .01		
Education	--	--	-0.03 [-0.057, 0.007]	< .05	-0.03 [-0.056, 0.007]	< .05		
Community size	--	--	-0.02 [-0.059, 0.012]	.08	-0.02 [-0.060, 0.013]	.10		
Political orientation	--	--	-0.01 [-0.062, 0.034]	.46	-0.02 [-0.081, 0.033]	.28		
Gini	--	--	--	--			0.65 [0.057, 1.250]	< .01
Deaths per million people	--	--	--	--			-0.07 [-0.524, 0.384]	.69
**Discretionary behaviors**								
Individualizing	0.32 [0.275, 0.371]	< .01	0.32 [0.268, 0.362]	< .01	0.29 [0.247, 0.325]	< .01		
Binding	-0.10 [-0.154, -0.049]	< .01	-0.10 [-0.150, -0.041]	< .01	-0.05 [-0.082, -0.013]	< .01		
Trust in government	0.09 [0.046, 0.136]	< .01	0.09 [0.051, 0.133]	< .01	0.10 [0.058, 0.135]	< .01		
Trust in citizens	0.12 [0.081, 0.163]	< .01	0.12 [0.074, 0.164]	< .01	0.12 [0.073, 0.166]	< .01		
Trust in science	0.17 [0.131, 0.206]	< .01	0.17 [0.126, 0.205]	< .01	0.16 [0.121, 0.197]	< .01		
Age	--	--	0.04 [0.003, 0.086]	< .01	0.05 [0.005, 0.091]	< .01		
Gender	--	--	0.03 [-0.006, 0.057]	< .05	0.03 [-0.005, 0.060]	< .05		
Education	--	--	0.02 [-0.012, 0.056]	.10	0.02 [-0.013, 0.056]	.10		
Community size	--	--	0.01 [-0.020, 0.048]	.28	0.02 [-0.012, 0.051]	.12		
Political orientation	--	--	-0.05 [-0.115, 0.017]	.06	-0.13 [-0.197, -0.065]	< .01		
Gini	--	--	--	--			-0.43 [-0.979, 0.115]	< .05
Deaths per million people	--	--	--	--			-0.19 [-0.647, 0.260]	.27

In step 2, the inclusion of individual level control variables (age, gender, education, community size, and political orientation) did not change the overall pattern of results, and associations between control variables and both the supposed mediators and independent variables were generally trivial in size (see Table 1). In step 3, the same mediation model was tested, this time with the inclusion of relevant country-level factors. Specifically, higher inequality (via the Gini index) was negatively associated with trust in government, citizens and science. Increased inequality was also positively associated with endorsement of prescribed behaviors, and negatively associated with endorsement of discretionary behaviors. The number of recorded deaths within each country (per million people) was positively associated with increased trust in science.

We also found evidence for a significant relationship between individualizing moral foundation and prescribed and discretionary behaviors via trust in citizen and science; furthermore, we found evidence for a significant relationship between the binding moral foundation and prescribed and discretionary behaviors via trust in government citizen and science (see Table 2 for indirect effects parameters and associated *p* values). These patterns showed that endorsement of individualizing moral foundations was associated to both prescribed and discretionary behavioral intentions via trust in science and, to a lesser extent, via trust in citizens. Endorsement of the binding moral foundations was indirectly related to both prescribed and discretionary behavioral intentions via trust in government and to a lesser extent via trust in citizens, and has a negative indirect relationship via trust in science.

**Table 2:** Indirect effects of the model depicted in Fig 1 when controlling for both the individual and country level variables.

Predictor	Mediator	Outcome	B	P
Individualizing MF	Trust in government	Prescribed behaviors	-0.001	.821
Individualizing MF	Trust in citizens	Prescribed behaviors	-0.003	< .05
Individualizing MF	Trust in science	Prescribed behaviors	0.047	< .01
Binding MF	Trust in government	Prescribed behaviors	0.03	< .01
Binding MF	Trust in citizens	Prescribed behaviors	-0.01	< .01
Binding MF	Trust in science	Prescribed behaviors	-0.025	< .01
Individualizing MF	Trust in government	Discretionary behaviors	-0.001	.819
Individualizing MF	Trust in citizens	Discretionary behaviors	0.005	< .01
Individualizing MF	Trust in science	Discretionary behaviors	0.031	< .01
Binding MF	Trust in government	Discretionary behaviors	0.025	< .01
Binding MF	Trust in citizens	Discretionary behaviors	0.017	< .01
Binding MF	Trust in science	Discretionary behaviors	-0.017	< .01

## Discussion

The COVID-19 pandemic represents the biggest health crisis of the last few decades. Addressing this crisis requires medical measures aimed at preventing and containing the virus. The pandemic has reshaped social life and social and behavioral scientists are committed to address these changes by studying how the virus is perceived, as well as its emotional implications. However, at a time when several vaccines have been released but the vast majority of the population around the world still need to be inoculated, it is all the more important to understand the social and psychological factors that motivate people to adopt individual behaviors aimed at enhancing the effectiveness of protective community measures. In the present research, we considered individuals’ intentions to comply with prescribed and discretionary behaviors to manage the spread of COVID-19 in 23 countries and examined the factors that might drive such behavioral intentions. We showed that the publicized statistics of the pandemic in terms of infections and deaths in each country does not represent the only, or even the most important antecedent of individual reactions, as a threat account of the health emergency would suggest [15,54]. In fact, the actual threat posed by the virus only accounted for a small percentage of variance in compliance with prescribed behaviors and did not at all predict intention to engage in discretionary efforts. Instead, considering psychological differences in terms of trust toward different agents—governments, citizens, and science—provide a more informative picture of individuals’ reactions to COVID-19.

Our results further show that the bases on which people form their moral judgments, that is, their moral foundations, might represent a key antecedent (among potential others) of trust that people devote to relevant agents (i.e., governments, citizens, and scientists). Interestingly, the endorsement of the binding foundations (i.e., loyalty, authority and purity) was positively related to trust in government and in citizens, but negatively related to trust in science. In contrast, endorsement of the individualizing foundations, which focuses on care and fairness, was negatively related to trust in institutions, and positively related to trust in science. Importantly, this was the case in particular with regards to discretionary behaviors, not just when evaluating compliance with prescribed measures. This is also consistent with the relationship between conservative ideology and distrust in science, which often takes the form of conspiracy theories [34].

Despite the promising results of this study, it should be acknowledged that findings are based on cross-sectional data that prevents us from making causal claims. Thus, future research should confirm the reported insights by using experimental designs to allow for causal inferences regarding the effects of morality and trust on societal behaviors. In a similar vein, the present research focused on self-reported behavioral intentions rather than actual behaviors. Thus, future work should go beyond self-report measures and examine how perceptions of trust shape actual prescribed and discretionary behaviors. These limitations considered, our data offer a number of potential practical implications and suggest several insights to improve institutional communication. Considering that in many countries communication about the measures have relied on statistics conveying the extent and severity of the health threat, our results might have far reaching consequences for the dissemination of institutional communication and public discourse. Our findings across 23 countries consistently revealed that the bases of moral reasoning differently shape the levels of trust that individuals place toward institutions, fellow citizens and, above all, in science. Despite the national, political, and cultural differences among the 23 countries studied in the study, as well as the varied severity of the COVID pandemic in terms of infections and deaths, the relationships between moral foundations, trust and COVID-related behaviors appeared consistent across these highly divergent contexts. Moreover, the three facets of trust we examined (directed to governments, fellow citizens, and science) emerged as key factors in shaping positive and prosocial behaviors that are crucial to limit the spread of the virus.

These results could further inform institutional and public communication in devising tailored messages or strategies designed to limit the spread of COVID-19. One might presume that in countries where individualizing moral foundations are prioritized over the binding foundations, communication based on trust in science and scientists would be the most pertinent on the modeling of individual behaviors. Conversely, in countries where binding moral pillars are more relevant, trust-based communication towards institutions may be more effective. Taken together, these findings suggest that communication strategies should consider the specifics of each country. Moreover, it would be key to tailor social intervention efforts to reflect the broader moral codes of a given country. Thus, multiple messages are needed to appeal to different subgroups of people, because it is unlikely that communications will change people’s moral foundations, but it is also unlikely that a single message will be equally effective in reaching all people. So, communications should not just target different groups in terms of demographics, but also consider that different subgroups within a population may care about different concerns and may have different reasons (not) to comply with requests to change their behaviors.

The present research further showed that trust in science represents a crucial factor in shaping individuals’ behavior in response to the COVID-19 pandemic. Although science should be a key source of public advice about behavioral strategies needed to face central topics for societal well-being, we have been witnessing a diffused distrust toward scientific advice, largely because of the spread of conspiracy theories and the subsequent tendencies to reject the official science. Regarding the spread of the COVID-19, a number of conspiracy theories about its origin, severity and prevention has been circulating, including false claims about the theory that COVID-19 vaccines are being used alter a person’s DNA, or about the fact that Big Pharma has a profit motive to exaggerate the benefits of vaccinations [e.g., 55]. Thus, enhancing trust in science—and fighting conspiracy theories—is crucial to promote the behavioral change that is needed to face the spread of the COVID-19. Our findings suggest that tailored messages could be key to induce such a behavioral change via the acceptance of scientific communication. Consistent with this view, recent finding showed the importance of trust in science both interacting with personal concerns and mediating between political conservatism and intellectual conspiracy thinking—among others—on behaving under the guidelines proposed by scientists [8].

Previous research has revealed that the perceived threat of disease might have negative implications for social relations. For instance, contagion enhances xenophobia, ethnocentrism, and discrimination [56,57]. Our study further departs from this evidence showing that trust might represent a buffer to counter these negative tendencies and, in stark contrast, to increase pro-sociality. This is particularly evident from our measures of discretionary behaviors, which are not prescribed by institutions, but by their very essence foster the well-being of the community at large, especially in this time of crisis. In conclusion, our data reveal that addressing a pandemic poses moral challenges, and taking advantage of individual (and cross-cultural) differences in moral principles that people endorse could play an important role in defeating COVID-19.

## Supporting information

S1 File (DOCX)
