# Supplementary material for: Trust predicts COVID-19 prescribed and discretionary behavioral intentions in 23 countries
Source: PLoS One. 2021 Mar 10;16(3):e0248334. doi: 10.1371/journal.pone.0248334 (PMC7946319; doi:10.1371/journal.pone.0248334)
Supplement: S1 File — (DOCX) [file pone.0248334.s001.docx]

**Supplementary materials**

*All materials and procedure*

The study was pre-registered at <https://osf.io/c4k2g/>. Data were collected between April, 10 and May, 19 2020. At the very beginning, participants provided their informed consent together with socio-demographic and personal information about Covid-19 symptoms. Then they were presented with the Moral Foundations Questionnaire (30 items; α = .85; Graham et al., 2011) and the Individualism-Collectivism scale (16 items; α_ind_ = .76; α_coll_ = .85; Triandis, McCusker, & Hui, 1990). After that, we presented participants with one out of two messages that conveyed the same content about the necessity to adhere to the COVID-19 prevention measures of social distancing, but with a different framing (appeal to individualizing vs. binding moral foundation). The two versions were similar in length, wording and structure. Participants were randomly assigned to one of the two experimental condition. To ascertain that the texts were balanced and the manipulation effective, we asked participants two questions about the valence of the article and five questions about its relatedness with each of the moral foundations. The message did not influence participants responses to the main DVs as anticipated, so this was not included in the subsequent analyses. The results of the pre-registered hypotheses are fully descripted on OSF (see Table S4 for the full description of the t-tests).

Participants then reported their trust toward the local institutions (2 items; e.g., *I trust our Government's competence in the management of the COVID-19 crisis;* α= .91), toward the other citizens (i.e., *I trust that other [country] citizens will respect the prescriptions imposed to avoid contagion*), and toward science (4 items, e.g., *We should trust the work of scientists*; α = .73). After that, they completed two ad-hoc created scales about their willingness to comply with the prescribed behaviors (7 items, e.g., *Whenever it is possible, self-isolating at home;* α = .82), as well as their intention to display discretionary behaviors (7 items, e.g., *According to our own possibility, all of us should give money to the charities to help them fight the disease;* α = .68) related to the management of the pandemic. the ingroup glorification scale (16 items; α = .94; Roccas, Klar, & Liviatan, 2006) and the short version of the RWA scale (10 items; α = .82; Roccato & Russo, 2015). Participants responded all questions on a 7-point scale from 1 (*not at all*) to 7 (*very much*). At the end of the questionnaire, they were fully debriefed. The survey was translated in the local languages. The complete materials and dataset may be found in the supplementary files. The complete data set is available at https://osf.io/c4k2g/.

**Table S1: Socio-demographic information by country**

| Country | Age | Gender | Type of community | N cohabitants | Education level | Political orientation | Symptoms of COVID-19 | Symptoms of COVID-19 (relatives/ friends) |
| --- | --- | --- | --- | --- | --- | --- | --- | --- |
| Argentina | 41.05(15.48) | W=182; M=78 | L=175; M=59; S=26 | 2.20(1.37) | 2.78(1.51) | 3.62(1.68) | Y=3; N=247; O=10 | Y=17; N=230; O=13 |
| Australia | 31.87(11.27) | W=130; M=169; O=4 | L=81; M=165; S=48; R=9 | 3.11(1.49) | 4.49(1.12) | 3.22(1.48) | Y=14; N=266; O=23 | Y=32; N=247; O=24 |
| Bangladesh | 30.43(5.73) | W=95; M=207; O=2 | L=210; M=18; S=46; R=30 | 4.60(2.42) | 4.11(.67) | 4.05(1.78) | Y=9; N=266; O=29 | Y=33; N=235; O=36 |
| Bosnia Hz | 29.49(10.42) | W=91; M=141; O=6 | L=156; M=29; S=20; R=33 | 3.36(1.53) | 2.74(.83) | 2.87(1.74) | Y=2; N=219; O=17 | Y=7; N=209; O=22 |
| Chile | 27.52(11.31) | W=223; M=81; O=10 | L=192; M=45; S=66; R=7 | 3.42(1.46) | 3.31(1.05) | 3.33(1.43) | Y=18; N=263; O=33 | Y=42; N=240; O=32 |
| China | 22.51(5.63) | W=254; M=121; O=20 | L=197; M=18; S=145; R=27 | 3.22(1.45) | 3.38(.85) | 3.54(1.16) | Y=4; N=385; O=6 | Y=1; N=388; O=5 |
| Finland | 51.49(18.43) | W=142; M=156; O=2 | L=155; M=39; S=73; R=33 | 2.59(1.36) | 3.45(1.25) | 4.22(1.45) | Y=1; N=258; O=31 | Y=28; N=238; O=34 |
| France | 45.52(15.25) | W=167; M=69; O=3 | L=79; M=44; S=50; R=62 | 2.79(1.93) | 4.03(1.36) | 3.15(1.61) | Y=18; N=188; O=33 | Y=92; N=117; O=30 |
| Germany | 28.14(10.52) | W=237; M=110; O=5 | L=69; M=61; S=208; R=14 | 1.85(1.24) | 3.34(1.50) | 3.04(1.19) | Y=15; N=287; O=50 | Y=68; N=254; O=30 |
| Greece | 28.04(8.38) | W=116; M=180; O=3 | L=172; M=43; S=48; R=36 | 3.64(1.35) | 3.72(1.22) | 3.31(1.34) | Y=12; N=265; O=22 | Y=40; N=234; O=25 |
| Ireland | 41.16(29.71) | W=227; M=89 | L=73; M=69; S=106; R=67 | 3.12(1.37) | 3.77(1.16) | 3.28(1.35) | Y=12; N=290; O=13 | Y=53; N=249; O=13 |
| Italy | 29.37(9.78) | W=124; M=226 | L=86; M=35; S=208; R=21 | 3.27(1.43) | 2.97(1.15) | 3.36(1.42) | Y=4; N=311; O=35 | Y=54; N=262; O=34 |
| Malaysia | 28.61(12.95) | W=126; M=50; O=3 | L=69; M=84; S=25; R=1 | 3.64(1.98) | 3.60(1.01) | 3.37(1.21) | Y=3; N=173; O=3 | Y=9; N=166; O=4 |
| Netherlands | 27.55(8.60) | W=117; M=199; O=4 | L=169; M=35; S=108; R=8 | 2.46(1.54) | 3.79(1.03) | 3.28(1.48) | Y=35; N=205; O=79 | Y=92; N=158; O=70 |
| Poland | 38.57(10.24) | W=139; M=173; O=2 | L=100; M=9; S=144; R=61 | 3.03(1.37) | 4.26(1.13) | 3.73(1.41) | Y=4; N=295; O=15 | Y=14; N=278; O=22 |
| Romania | 41.65(11.95) | W=200; M=181 | L=200; M=7; S=105; R=69 | 2.90(1.23) | 3.54(1.34) | 3.49(1.75) | Y=14; N=342; O=25 | Y=26; N=329; O=26 |
| Russia | 44.12(11.23) | W=199; M=117; O=1 | L=233; M=40; S=33; R=11 | 2.79(1.35) | 4.47(1.02) | 3.85(1.89) | Y=12; N=290; O=13 | Y=53; N=249; O=13 |
| SKorea | 37.42(14.75) | W=81; M=48; O=1 | L=80; M=32; S=16; R=2 | 3.14(1.36) | 3.93(1.05) | 3.61(1.47) | Y=3; N=121; O=6 | Y=10; N=115; O=5 |
| Spain | 37.23(13.26) | W=248; M=68; O=4 | L=51; M=16; S=235; R=18 | 2.65(1.31) | 3.85(1.16) | 2.75(1.60) | Y=13; N=268; O=39 | Y=70; N=220; O=30 |
| Switzerland | 42.29(15.82) | W=240; M=101; O=5 | L=73; M=48; S=128; R=93 | 2.87(1.41) | 4.09(1.36) | 3.05(1.34) | Y=20; N=276; O=49 | Y=102; N=204; O=39 |
| Turkey | 23.07(6.47) | W=203; M=95; O=2 | L=242; M=25; S=32; R=1 | 4.00(1.25) | 2.51(.95) | 3.33(1.16) | Y=21; N=251; O=28 | Y=46; N=219; O=35 |
| UK | 32.63(11.49) | W=214; M=83; O=3 | L=68; M=42; S=149; R=41 | 2.58(1.43) | 3.44(1.20) | 3.34(1.49) | Y=30; N=245; O=25 | Y=87; N=194; O=19 |
| US | 31.53(13.78) | W=233; M=121; O=5 | L=106; M=152; S=76; R=25 | 2.66(1.75) | 3.45(.94) | 3.09(1.62) | Y=17; N=326; O=16 | Y=57; N=278; O=24 |

Note: Gender: M=man, W=woman, O = not declare/non binary; Type of community: L=large city, M=suburb near a large city, S=small city, R=rural area; Education level: scale: 1 (low) - 6 (high); Political orientation: scale 1 (left-wing/liberal) - 7 (right-wing/conservative); Symptoms of COVID-19: Y = Yes; N = NO; O = Unsure/I don't know exactly what the symptoms of Covid-19 are

**Table S2: Descriptive statistics for the main variables by country**

| Country | Collectivism | Individualism | MF  individualizing | MF  binding | RWA | Trust in istitutions | Trust in citizens | Trust in science | Covid19  prescribed | Covid19  discret. | Ingroup glorific. |
| --- | --- | --- | --- | --- | --- | --- | --- | --- | --- | --- | --- |
| Argentina | 5.43(.99) | 4.26 (.95) | 5.80 (.87) | 4.32 (1.02) | 2.80 (.95) | 4.61 (1.77) | 3.71 (1.43) | 5.60 (1.14) | 6.64 (.76) | 4.80 (1.09) | 4.46 (1.21) |
| Australia | 4.96 (.91) | 4.69 (.78) | 5.39 (.72) | 3.84 (1.00) | 2.83 (.99) | 4.34 (1.53) | 4.04 (1.52) | 5.45 (1.00) | 6.13 (.73) | 4.92 (.99) | 3.92 (1.25) |
| Bangladesh | 5.16 (.72) | 4.61 (.72) | 5.33 (.82) | 5.02 (.77) | 5.11 (.75) | 3.55 (1.97) | 4.52 (1.71) | 5.22 (1.09) | 6.49 (.77) | 5.74 (1.13) | 5.28 (.92) |
| Bosnia Hz | 5.46 (.96) | 5.04 (.93) | 5.83 (.81) | 4.78 (1.02) | 3.75(1.15) | 2.68 (1.52) | 3.99 (1.81) | 4.43 (1.45) | 5.91 (1.34) | 4.90 (1.21) | 3.10 (1.47) |
| Chile | 5.25 (.86) | 4.14 (.78) | 5.83 (.62) | 3.70 (.83) | 2.44 (.90) | 2.40 (1.64) | 2.72 (1.36) | 4.99 (1.15) | 6.68 (.57) | 4.97 (.93) | 3.22 (1.19) |
| China | 4.51 (.97) | 4.77 (.96) | 4.45 (.93) | 4.27 (.92) | 3.85 (.63) | 5.73 (1.25) | 4.36 (1.63) | 5.05 (.83) | 5.91 (1.04) | 4.62 (1.04) | 5.13 (1.18) |
| Finland | 4.76 (.87) | 4.09 (.87) | 5.05 (.94) | 4.39 (.84) | 3.52 (.95) | 5.09 (1.47) | 4.51 (1.33) | 5.18 (.94) | 5.81 (.95) | 4.39 (.95) | 4.82 (1.11) |
| France | 5.12 (.79) | 4.19 (.88) | 5.39 (.78) | 3.79 (.85) | 2.64(1.00) | 3.20 (1.82) | 3.95 (1.57) | 5.22 (1.12) | 6.10 (.71) | 5.01 (1.03) | 3.88 (1.11) |
| Germany | 4.96 (.83) | 4.18 (.79) | 5.63 (.64) | 3.65 (.75) | 2.29 (73) | 5.09 (1.35) | 4.55 (1.42) | 5.77 (.92) | 5.88 (.83) | 5.53 (.84) | 2.85 (.85) |
| Greece | 4.69 (.83) | 4.48 (.82) | 5.49 (.72) | 4.50 (.85) | 3.04 (.99) | 4.54 (1.68) | 3.88 (1.54) | 5.65 (.92) | 6.09 (.82) | 5.01 (.86) | 3.54 (1.25) |
| Ireland | 5.32 (.87) | 4.43 (.89) | 5.57 (83) | 4.14 (.98) | 2.86 (.92) | 5.06 (1.58) | 4.50 (1.49) | 4.98 (1.19) | 6.13 (.83) | 5.24 (95) | 4.47 (.07) |
| Italy | 5.53 (.75) | 4.55 (.80) | 5.57 (.70) | 4.25 (.82) | 2.98 (.99) | 4.82 (1.54) | 3.91 (1.48) | 5.47 (.98) | 6.40 (.69) | 5.05 (.87) | 4.48 (1.09) |
| Malaysia | 5.29 (76) | 4.78 (82) | 5.48 (.64) | 4.41 (.84) | 3.68 (.87) | 5.02 (1.19) | 4.51 (1.42) | 4.98 (.85) | 6.25 (.74) | 5.37 (.73) | 4.46 (1.02) |
| Netherlands | 4.59 (.74) | 4.58 (.74) | 5.31 (.66) | 3.89 (.79) | 2.71 (.90) | 5.08 (1.26) | 4.53 (1.29) | 5.54 (.86) | 5.75 (.66) | 4.90 (.94) | 3.65 (.90) |
| Poland | 5.56 (.99) | 4.77 (.76) | 5.71 (.71) | 4.41 (1.02) | 3.13(1.07) | 2.95 (1.77) | 4.58 (1.51) | 5.20 (1.22) | 6.07 (.95) | 4.86 (1.09) | 3.79 (1.22) |
| Romania | 5.62 (.92) | 5.28 (.90) | 5.44 (.91) | 4.80 (.95) | 4.20(1.10) | 3.60 (1.90) | 3.77 (1.74) | 4.66 (1.23) | 6.27 (1.15) | 4.94 (1.21) | 4.64 (1.32) |
| Russia | 4.91 (.96) | 4.82 (.84) | 5.13 (97) | 4.46 (1.10) | 3.85(1.50) | 3.67 (2.17) | 3.66 (1.71) | 5.13 (1.28) | 6.06 (1.13) | 4.62 (1.21) | 4.29 (1.66) |
| SKorea | 4.68 (1.00) | 4.78 (79) | 5.06 (.80) | 4.09 (.88) | 3.77 (.93) | 6.49 (1.60) | 6.13 (1.37) | 5.22 (1.02) | 6.16 (.83) | 4.63 (1.03) | 4.25 (1.03) |
| Spain | 5.41 (.77) | 4.13 (.85) | 5.85 (.69) | 3.82 (.84) | 2.26 (.78) | 3.99 (1.96) | 4.24 (1.56) | 5.72 (.93) | 6.40 (.71) | 5.22 (.95) | 3.94 (1.17) |
| Switzerland | 5.01 (.90) | 4.15 (.85) | 5.56 (.80) | 3.68 (.97) | 2.33 (.91) | 4.74 (1.70) | 4.42 (1.41) | 5.23 (1.13) | 5.67 (1.04) | 5.21 (.96) | 3.89 (1.17) |
| Turkey | 5.20 (.92) | 4.90 (.85) | 5.67 (.72) | 3.98 (.97) | 3.00(1.01) | 3.53 (1.73) | 2.40 (1.52) | 5.22 (1.01) | 6.55 (.66) | 4.98 (1.00) | 3.97 (1.32) |
| UK | 5.06 (.87) | 4.58 (.76) | 5.41 (.74) | 4.16 (.86) | 3.06 (.98) | 4.19 (1.76) | 3.89 (1.57) | 5.14 (1.04) | 6.28 (.63) | 5.17 (.94) | 3.97 (1.33) |
| US | 5.08 (94) | 4.66 (.87) | 5.47 (.77) | 3.95 (1.11) | 2.95(1.11) | 3.00 (1.74) | 3.15 (1.65) | 5.14 (1.21) | 6.19 (.99) | 5.20 (1.08) | 3.66 (1.51) |

**Table S3. Residual variances of estimated parameters of the three models tested**

|  | Step 1 | | Step 2 | | Step 3 | | | |
| --- | --- | --- | --- | --- | --- | --- | --- | --- |
|  |  |  |  |  | *Within Level* | | *Between Level* | |
|  | β | *p* | β | *p* | β | *p* | β | *p* |
| Trust in government | 0.93 | < .01 | 0.926 | < .01 | 0.933 | < .01 | 0.496 | < .01 |
| Trust in citizens | 0.968 | < .01 | 0.957 | < .01 | 0.958 | < .01 | 0.332 | < .01 |
| Trust in science | 0.963 | < .01 | 0.957 | < .01 | 0.96 | < .01 | 0.346 | .057 |
| Prescribed behaviors | 0.828 | < .01 | 0.828 | < .01 | 0.828 | < .01 | 0.21 | < .05 |
| Discretionary behaviors | 0.81 | < .01 | 0.81 | < .01 | 0.807 | < .01 | 0.241 | < .05 |

**Table S4: T-tests relative to the effect of the message framing (Individualizing vs. Binding moral foundation) on the main DVs of the study.**

| Dependent variable | Individualizing message condition (M and SD) | Binding message condition (M and SD) | *t* | *p* |
| --- | --- | --- | --- | --- |
| Trust in institutions | 4.22 (1.93) | 4.18 (1.92) | 0.76 | .45 |
| Trust in citizens | 4.01 (1.67) | 4.04 (1.66) | -0.73 | .47 |
| Trust in science | 5.23 (1.11) | 5.24 (1.12) | -0.24 | .81 |
| Prescribed Behaviors | 6.18 (0.89) | 6.14 (0.93) | 1.45 | .15 |
| Discretionary Behaviors | 5.02 (1.05) | 5.03 (1.06) | -.32 | .75 |

**References**

Roccas, S., Klar, Y., & Liviatan, I. (2006). The paradox of group-based guilt: modes of national identification, conflict vehemence, and reactions to the in-group's moral violations. *Journal of Personality and Social Psychology, 91*(4), 698. DOI: 10.1037/0022-3514.91.4.698

Roccato, M., & Russo, S. (2015). Two short, balanced, unidimensional, invariant across genders parallel forms of Altemeyer’s (1996) Right-Wing Authoritarianism Scale. *Psicologia Sociale, 10*(3), 257–272. DOI: 10.1482/81371.

Triandis, H. C., McCusker, C., & Hui, C. H. (1990). Multimethod probes of individualism and collectivism. *Journal of Personality and Social Psychology, 59*(5), 1006.
